# A Mild Case of Autoimmune Glial Fibrillary Acidic Protein Astrocytopathy With Chronic Onset

**DOI:** 10.7759/cureus.83926

**Published:** 2025-05-11

**Authors:** Kenta Aohara, Keitaro Kurooka, Masafumi Nishikawa, Itsuki Hasegawa, Akitoshi Takeda, Hiroshi Tsuji, Yoshiaki Itoh

**Affiliations:** 1 Department of Neurology, Osaka Metropolitan University, Osaka, JPN; 2 School of Medicine, Osaka Metropolitan University, Osaka, JPN

**Keywords:** autoimmune glial fibrillary acidic protein astrocytopathy, chronic meningitis, diffuse leukoencephalopathy, hand tremor, optic disc swelling, smodering

## Abstract

A 25-year-old woman presented with mild fever and fatigue that began four months prior to admission. At 30 days after onset, she developed bilateral visual field impairment with hand tremors. Gait difficulty occurred at 60 days after onset. On admission, the patient was alert and well-oriented. Examination revealed central scotomas, papilledema in both eyes, postural tremors in both hands, hyperreflexia in the limbs, positive pathological reflexes in the lower extremities, and spastic gait. Cerebrospinal fluid analysis showed an elevated cell count and protein levels as well as positive glial fibrillary acidic protein (GFAP) α antibodies. Brain magnetic resonance imaging (MRI) revealed hyperintensities in the bilateral cerebral hemispheres and dorsal brainstem on T2-weighted images. These lesions did not show enhancement with gadolinium. Methylprednisolone pulse therapy followed by oral prednisolone led to almost complete resolution of neurological symptoms and MRI abnormalities. Although GFAP astrocytopathy typically presents acutely or subacutely with severe symptoms, it should be considered when patients present with a more chronic course and relatively mild symptoms, as seen in this case. GFAP astrocytopathy should also be considered when patients present with gradually expanding white matter lesions.

## Introduction

Autoimmune glial fibrillary acidic protein (GFAP) astrocytopathy is a neurological disorder characterized by the presence of autoantibodies targeting GFAP, an intermediate filament protein expressed in astrocytes, leading to meningoencephalitis or meningoencephalomyelitis [1].

The estimated prevalence of GFAP astrocytopathy is 0.6 per 100,000 population, with an annual incidence of 0.03 per 100,000 [2].

Although the pathogenic mechanism of GFAP antibodies remains incompletely elucidated, histopathological analyses frequently reveal pronounced perivascular inflammation, particularly T-cell infiltration surrounding small vessels in the brain and spinal cord. These findings suggest that antigen-specific T-cell responses directed against GFAP may play a central role in disease pathogenesis. While GFAP antibodies are considered a highly specific diagnostic biomarker, it remains uncertain whether they are directly pathogenic or represent an epiphenomenon secondary to T-cell-mediated autoimmunity [1,3,4].

Clinically, most reported cases demonstrate an acute or subacute onset, often presenting with meningeal symptoms such as headache and fever, encephalitic features including cognitive impairment and seizures, and additional manifestations depending on lesion localization, such as motor paralysis attributable to myelitis. Autonomic dysfunction is also frequently reported. Furthermore, approximately 40% of patients experience a preceding influenza-like prodrome, although no specific infectious agent has been definitively identified [5]. Proposed immunopathological mechanisms include molecular mimicry, wherein viral antigens resemble GFAP and incite an autoimmune response, and bystander activation, whereby CNS inflammation exposes self-antigens and disrupts immune tolerance.

On brain magnetic resonance imaging (MRI), gadolinium-enhanced scans often demonstrate characteristic linear periventricular enhancement, a hallmark imaging feature of this disease [3-6].

Herein, we report an atypical case of autoimmune GFAP astrocytopathy with a chronic clinical course and relatively mild symptomatology. Despite the absence of classic encephalitic signs such as altered consciousness or seizures, the patient exhibited visual disturbances, pyramidal tract signs, and tremors.

## Case presentation

**Figure 1:**
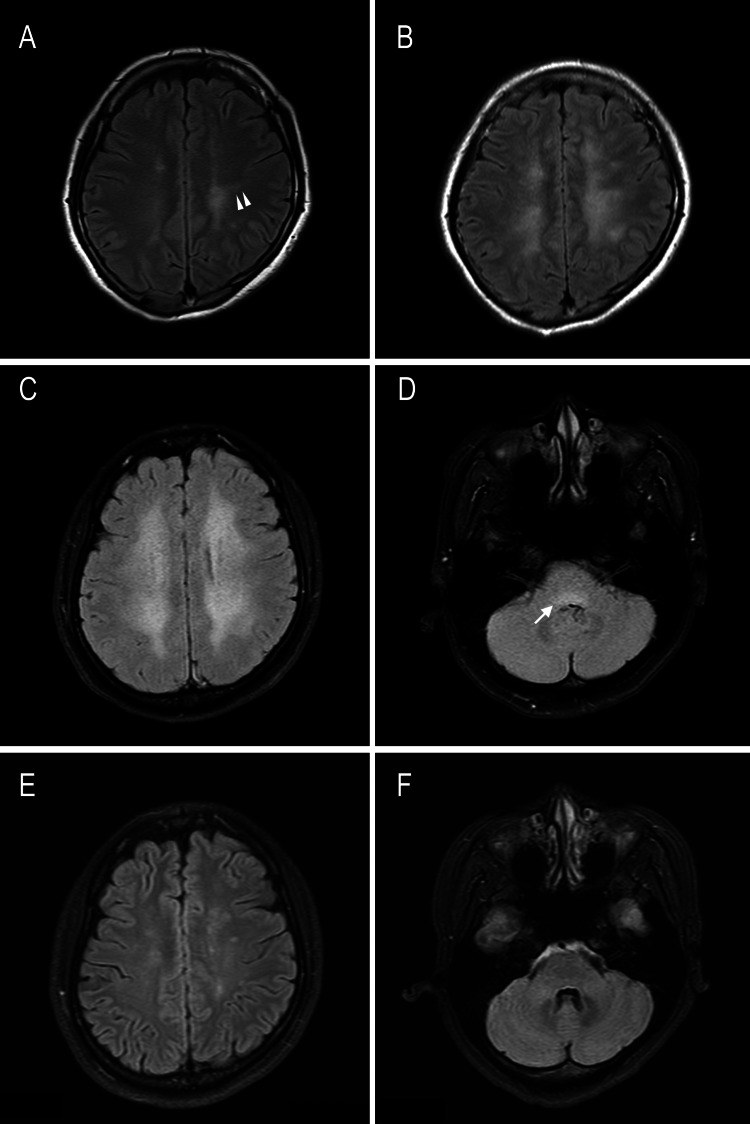
Findings of serial plain fluid-attenuated inversion recovery (FLAIR) MRI of the brain throughout treatment (A) Brain image obtained using MRI 30 days after onset showing high-intensity signals in the periventricular white matter on FLAIR images that appear as radiating and linear hyperintense lesions (arrowheads). (B) Another image obtained using MRI 30 days after onset revealing enlargement of the lesions surrounding the cerebral ventricles. (C, D) Brain images obtained using MRI at admission and 130 days after onset demonstrating further expansion of the white matter lesions and new hyperintense areas in the dorsal brainstem (arrow). (E, F) Images obtained using MRI after treatment and 150 days after onset showing reduced periventricular white matter lesions and resolution of the dorsal brainstem lesions.

A 25-year-old woman with right-hand dominance and a history of childhood asthma and dysmenorrhea presented with visual field defects and hand tremors. Four months prior to admission, she experienced mild fever and fatigue; subsequently, she experienced bilateral visual deficits and hand tremors 30 days later. Brain MRI without Gd contrast performed at another hospital revealed bilateral white matter lesions that appeared as radiating and linear hyperintense areas on fluid-attenuated inversion recovery (FLAIR) images (Figure 1A). Despite the absence of definitive MRI findings at the previous hospital, cerebral venous sinus thrombosis was diagnosed and she was prescribed warfarin as an anticoagulant. Despite slight vision improvement, she developed gait difficulty 60 days after symptom onset. Additional MRI performed 90 days after symptom onset showed progression of the white matter lesions (Figure 1B). Subsequently, she was referred to our hospital for further evaluation. However, she refused admission until 130 days after symptom onset because she was busy with work and did not perceive her symptoms as serious.

**Figure 2:**
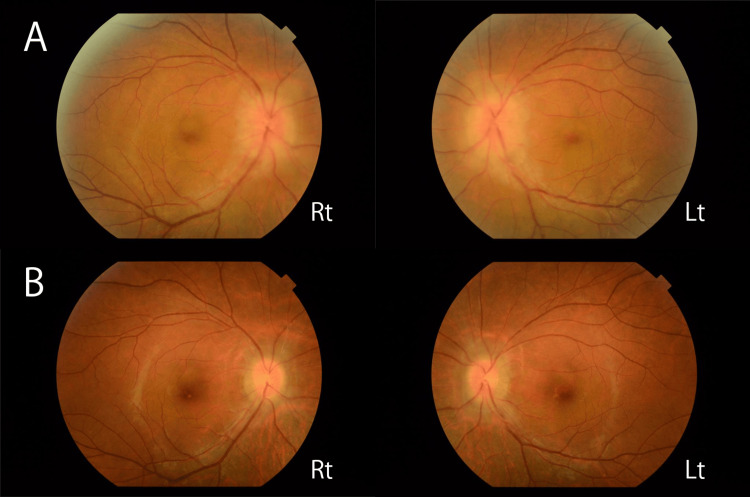
Fundus findings (A) Papilledema observed before treatment (130 days after onset). (B) Improved papilledema after treatment (150 days after onset).

An examination revealed the following: blood pressure, 99/77 mmHg; pulse, 84 bpm; SpO2, 96% on room air; and body temperature, 36.4°C. A neurological assessment revealed that the patient was conscious and well-oriented. The patient had a visual acuity of 20/25 in both eyes, accompanied by central scotomas. Fundoscopy revealed bilateral optic disc swelling without venous dilation, vascular tortuosity, or hemorrhage (Figure 2A).

Anisocoria was not observed, and the pupillary light reflex was rapid bilaterally. Eye movements were normal and without diplopia, and palatal elevation and uvular deviation were normal. Muscle atrophy was not noted, and muscle strength was preserved. Reflex testing showed hyperactive biceps and triceps reflexes, a more pronounced patellar tendon reflex on the right, positive Hoffmann and Trömner reflexes bilaterally, and a positive Babinski reflex on both sides. The Chaddock reflex was negative. Pseudoclonus was observed in both lower limbs. Temperatures and deep sensations remained normal. Coordination testing did not reveal ataxia; however, a 6-Hz resting tremor was present in both hands. A gait examination revealed mild spasticity, instability during single-leg standing, and an unsteady tandem gait; however, Romberg’s sign was negative. Bladder and rectal dysfunction were not observed. Meningeal irritation signs were not detected. The modified Rankin Scale (mRS) score was 2.

Laboratory tests showed a white blood cell count of 7,100/μL (reference range: 3,300-8,600/μL) and C-reactive protein (CRP) level of 0.02 mg/dL (reference range: 0.00-0.14 mg/dL). No significant inflammatory response was observed, and hyponatremia was not detected (Table 1). Serum aquaporin-4 (AQP4) antibodies were measured using an enzyme-linked immunosorbent assay (ELISA), which yielded negative results. Opening pressure during lumbar puncture was 8 cmH2O (reference: 7-18 cmH2O). A cerebrospinal fluid (CSF) analysis showed elevated protein levels (84 mg/dL, reference range: 10-40 mg/dL), a slightly increased cell count (13/μL; reference range: <5/μL; 100% mononuclear cells), an elevated immunoglobulin G (IgG) index (1.09; reference: <0.75), and mildly decreased glucose levels (44 mg/dL; reference: 50-75 mg/dL). Oligoclonal bands were detected (10 bands). Cultures for bacteria, fungi, and Mycobacterium tuberculosis in CSF indicated the absence of growth. The polymerase chain reaction (PCR) test for Mycobacterium tuberculosis also yielded negative results. A cytological examination of the CSF revealed no malignant cells. Based on the clinical symptoms at admission and CSF findings, an infectious etiology was considered unlikely; therefore, neither antiviral nor antibacterial agents were administered.

**Table 1:** Laboratory Parameters of the Serum and Cerebrospinal Spinal Fluid CRP: C-reactive protein, AQP4: aquaporin-4, CSF: cerebrospinal fluid, Ig: immunoglobulin, OCB: oligoclonal bands, MOG: myelin-oligodendrocyte glycoprotein antibody, NMDAR: N-methyl-D-aspartic acid receptor, GFAP: glial fibrillary acidic protein

	Laboratory parameters	Value (units)	Reference value
Serum	WBC	7100/μL	3300-8600/μL
	Sodium	138 mmol/L	138-145 mmol/L
	Glucose	95 mg/dL	73-109 mg/dL
	CRP	0.02 mg/dL	0-0.14 mg/dL
	AQP4 antibody	Negative	Negative
CSF	Cells	13/μL	<5/μL
	Lymphocytes	100%	
	Protein	84 mg/dL	10-40 mg/dL
	Glucose	44 mg/dL	50-75 mg/dL
	IgG index	1.09	<0.75
	OCB	Positive	Negative
	MOG antibody	Negative	Negative
	NMDAR antibody	Negative	Negative
	GFAP antibody	Positive	Negative

Anti-myelin-oligodendrocyte glycoprotein antibody and anti-N-methyl-D-aspartic acid receptor antibody in the CSF were measured using a cell-based assay, and both yielded negative results. Gifu University used transfected cell-based and tissue-based immunofluorescence assays to detect GFAPα antibodies in the CSF and noted positive results. The main test results are listed in Table 1.

**Figure 3:**
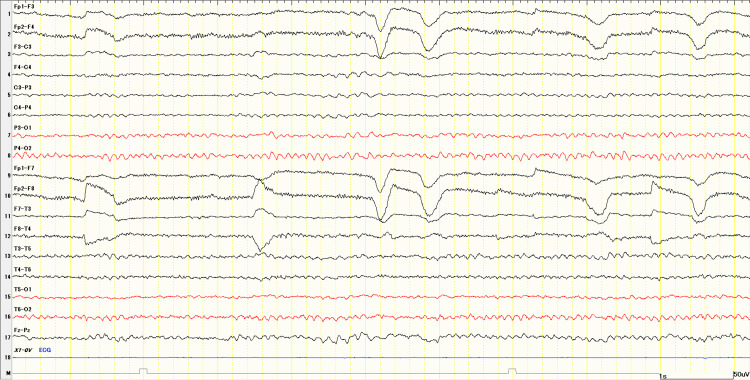
EEG recorded at admission. The basic rhythm was 9 to 11 Hz and 20 to 40 μV, predominantly in the occipital region. Abnormal discharges are not observed. EEG: electroencephalogram

Electroencephalography (EEG) showed a basic rhythm of 9 to 11 Hz and 20 to 40 μV, predominantly in the occipital region; epileptiform discharges were not observed (Figure 3).

**Figure 4:**
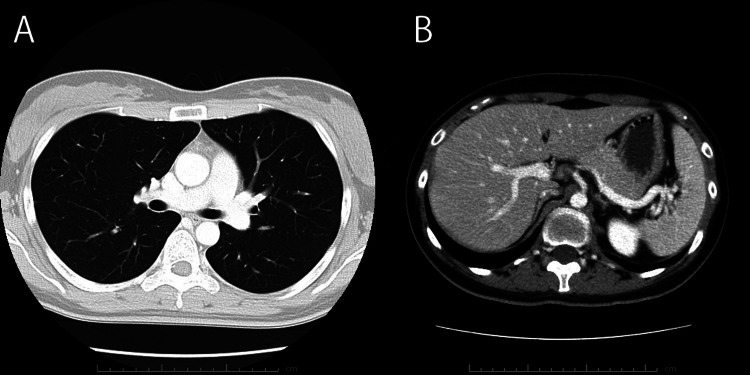
Enhanced chest and abdominal CT images at admission. (A) Chest CT image showing no lung lesions. (B) Abdominal CT image showing no abnormalities. CT: computed tomography

Chest and abdominal computed tomography revealed no abnormalities, thus ruling out paraneoplastic syndrome (Figure 4).

Brain MRI showed expanded hyperintense lesions in the white matter and dorsal brainstem (Figure 1C, 1D) without contrast enhancement 130 days after onset. MRI of the orbital area and spine yielded normal results. The central critical flicker fusion frequency in the right eye and that in the left eye were 25 and 22 Hz, respectively.

**Figure 5:**
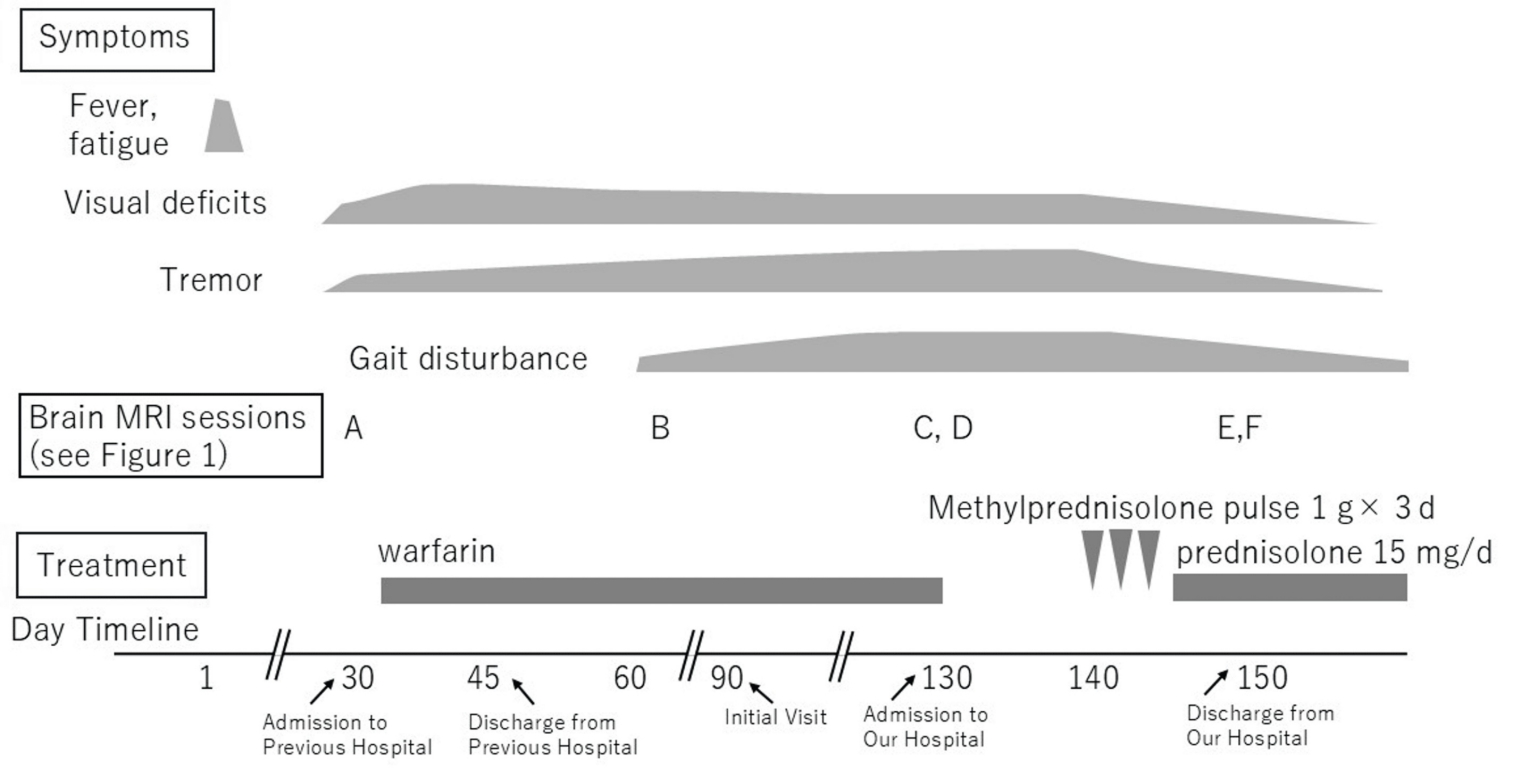
Clinical course of the patient.

Visual impairment, hand tremor, and characteristic imaging findings were observed. Ultimately, positive GFAP antibody results were observed, leading to a diagnosis of autoimmune GFAP astrocytopathy. Intravenous methylprednisolone pulse therapy (1.0 g/day for three days) was initiated 140 days after onset (Figure 5). Tremors improved following treatment. At five days after pulse therapy, a follow-up CSF analysis showed the same cell count (13/μL; reference range: <5/μL; 100% mononuclear cells) and improved protein levels (39 mg/dL). Follow-up MRI revealed regression of the white matter and dorsal brainstem lesions (Figure 1E, 1F) at 150 days after onset. Maintenance therapy with prednisolone (15 mg/d) was introduced, leading to the resolution of tremors and gait disturbances. Bilateral visual acuity improved to 20/20, and optic disc swelling resolved (Figure 2B). The central critical flicker fusion frequency increased to 40 Hz in the right eye and 34 Hz in the left eye. At discharge, the mRS score improved to 1.

## Discussion

We report a case of autoimmune GFAP astrocytopathy that initially presented with flu-like symptoms (mild fever and fatigue), followed by tremors, visual disturbances, and gait instability. This condition is a form of autoimmune meningoencephalitis, characterized by the presence of anti-GFAP antibodies in the CSF [1]. Flu-like symptoms occur in about 40% of patients, and optic disc swelling with blurred vision is seen in approximately 30% [5]. Our patient also exhibited hyperreflexia and tremors, both of which are frequently reported features (Table 2) [4-6].

**Table 2:** Comparison Between Typical Features of Autoimmune Glial Fibrillary Acidic Protein Astrocytopathy and Those of the Present Case ¹ Typical frequencies are based on the following previous studies: Kimura et al. [4], Flanagan et al. [5], and Gravier-Dumonceau et al. [6].

Category	Feature	Typical Frequency ^1^	Present Case
Onset	Acute or subacute onset	71%–81%	Chronic (≥130 days)
Clinical symptoms	Flu-like symptoms (fever, fatigue)	40%	Present
	Visual disturbance/papilledema	30%	Present
	Hyperreflexia	23%–68%	Present
	Tremor	40%–41%	Present
	Consciousness disturbance	50%–75%	Absent
	Seizures	11%–19%	Absent
	Bladder dysfunction	6%–71%	Absent
	Meningeal signs other than headache	32%–61%	Absent
Serum findings	Hyponatremia	15%–30%	Absent
CSF findings	Elevated protein	83%–98%	Present
	Pleocytosis (≥5/μL)	88%–99%	Present
	Oligoclonal bands	54%–77%	Present
MRI findings	Linear radial enhancement	32%–53 %	Absent
	Abnormal T2-weighted imaging/FLAIR hyperintensities	56%–83 %	Present
	Brainstem lesions	27%–31%	Present
Functional outcome	Modified Rankin Scale score at peak	≥3 in 82%–89%	2
	Modified Rankin Scale score at discharge	≥3 in 11%–30%	1

This case exhibited an atypical course with gradual and chronic progression over the course of more than 120 days between onset and hospitalization; however, the peak mRS score was 2, indicating relatively mild symptoms. In contrast, 71% to 81% of autoimmune GFAP astrocytopathy cases exhibit an acute to subacute course and progress within three months [1,5]. To our knowledge, only six chronic autoimmune GFAP astrocytopathy cases have been reported [7-10]. Two cases manifested drug-resistant epilepsy [7], whereas four cases manifested progressive cognitive decline for more than three months [8-10]. However, compared to the present case, all of the previously reported cases were more severe.

The most characteristic MRI finding is perivascular linear enhancement around the lateral ventricles, which is observed in 32 to 56% of patients (Table 1) [3-6]. The initial MRI showed radiating and linear hyperintense lesions (Figure 1A), but Gd contrast was not used. The radiating and linear hyperintense white matter lesions (Figure 1A) subsequently progressed to more confluent lesions (Figure 1B, 1C) resembling leukoencephalopathy, which complicated the diagnosis. Non-contrast FLAIR MRI of the brain showed multiple hyperintense lesions in the periventricular and deep white matter in 41% to 44% of cases [4,6]. However, to the best of our knowledge, no studies have examined how these lesions expand over the course of the disease. A key diagnostic feature was the presence of brainstem lesions (Figure 1C, 1D), which are seen in 31% of patients with autoimmune GFAP astrocytopathy [3,6].

Typical cases of GFAP astrocytopathy follow an acute to subacute clinical course, and brain MRI often reveals Gd-enhancing lesions. These findings suggest disruption of the blood-brain barrier and indicate high disease activity [1,3-6]. In contrast, the absence of enhancement in the present case may have implied lower disease activity. However, given the progressive expansion of white matter lesions and chronic worsening of gait disturbance, we interpreted this case as a smoldering and progressive subtype. Although the diagnosis was delayed, we believe that identifying the disease and initiating treatment before the symptoms were severely advanced contributed to the relatively mild clinical outcome.

## Conclusions

Our patient had an atypical case of autoimmune GFAP astrocytopathy with chronic progression; however, classical encephalitic features such as altered consciousness and seizures were not apparent. Visual disturbances, pyramidal signs, and tremors were observed. The patient’s clinical symptoms as well as gradually expanding white matter lesions without Gd enhancement may represent a smoldering type of autoimmune GFAP astrocytopathy that could potentially constitute a novel clinical subtype.

Recognition of the typical manifestations and imaging findings of autoimmune GFAP astrocytopathy, even in the absence of acute or severe encephalitis, is crucial to its diagnosis. Autoimmune GFAP astrocytopathy should be considered when patients present with chronically progressive neurological symptoms. Initiating appropriate treatment may help prevent severe disease progression. The accumulation of additional cases with a smoldering clinical course, as observed in the present case, is essential to furthering the understanding of the spectrum of this disease.
